# Unsupervised color image segmentation: A case of RGB histogram based K-means clustering initialization

**DOI:** 10.1371/journal.pone.0240015

**Published:** 2020-10-22

**Authors:** Sadia Basar, Mushtaq Ali, Gilberto Ochoa-Ruiz, Mahdi Zareei, Abdul Waheed, Awais Adnan

**Affiliations:** 1 Department of Information Technology, Hazara University, Mansehra, Pakistan; 2 Department of Computer Science, Abbottabad University of Science and Technology, Abbottabad, Pakistan; 3 Tecnologico de Monterrey, School of Engineering and Sciences, Zapopan, Mexico; 4 School of Electrical and Computer Engineering, Seoul National University, Seoul, South Korea; 5 Department of Computer Science, Institute of Management Sciences, Peshawar, Pakistan; COMSATS University Islamabad, Wah Campus, PAKISTAN

## Abstract

Color-based image segmentation classifies pixels of digital images in numerous groups for further analysis in computer vision, pattern recognition, image understanding, and image processing applications. Various algorithms have been developed for image segmentation, but clustering algorithms play an important role in the segmentation of digital images. This paper presents a novel and adaptive initialization approach to determine the number of clusters and find the initial central points of clusters for the standard K-means algorithm to solve the segmentation problem of color images. The presented scheme uses a scanning procedure of the paired Red, Green, and Blue (RGB) color-channel histograms for determining the most salient modes in every histogram. Next, the histogram thresholding is applied and a search in every histogram mode is performed to accomplish RGB pairs. These RGB pairs are used as the initial cluster centers and cluster numbers that clustered each pixel into the appropriate region for generating the homogeneous regions. The proposed technique determines the best initialization parameters for the conventional K-means clustering technique. In this paper, the proposed approach was compared with various unsupervised image segmentation techniques on various image segmentation benchmarks. Furthermore, we made use of a ranking approach inspired by the Evaluation Based on Distance from Average Solution (EDAS) method to account for segmentation integrity. The experimental results show that the proposed technique outperforms the other existing clustering techniques by optimizing the segmentation quality and possibly reducing the classification error.

## Introduction

Image segmentation splits an image into sub-regions where each region shares common properties among the pixels. It is used to find homogeneous regions of different objects based on certain properties such as texture, color intensity, and edge information [1, 2]. The image segmentation process yields a set of regions that can be distinctively separated in a meaningful way (which depends on the target application). In some scenarios, the segmentation process makes it easier to localize objects, extract image boundaries, and further details of objects in images [3]. The segmentation process is one of the most important stages in image analysis, computer vision, image understanding, and image compression as it reduces the complexity of the image and facilitates the work of other high-level processing tasks.

There are various types of segmentation algorithms based on region detection and extraction, edge detection, thresholding techniques [4, 5], physics-based schemes, and data clustering methods [2, 6–12]. The data clustering approach divides objects into different classes and subclasses, where the data points of the same class are similar but dissimilar from data points of other classes [13, 14]. Clustering is one of the most popular techniques for image segmentation, data analysis, and data mining [15]. Clustering approaches also play an important role in the medical domain for the early diagnosis of pulmonary nodules [16], Magnetic Resonance Imaging (MRI) [17], clustering of bipolar disorder [18] and automatic clustering algorithms for super-particles [19].

One of the simplest algorithms for clustering is the K-means algorithm that was introduced by MacQueen in 1967 [19]. The K-means algorithm works by dividing a database into k-groups [19–21]. The K-means method divides the dataset entered by users and collects the unmarked data points which are then distributed among K clusters, the object centroids identified by some certain pre-selected criterion about distance [22, 23]. According to the clustering criterion, the inter-cluster dissimilarity is increased while the intra-cluster distance is reduced [24]. In comparison to hierarchical clustering, the K-means algorithm is simple and computationally efficient [8, 25]. Four different schemes adopted for the initialization of K-value selection are: the Elbow algorithm, Gap Statistic, Canopy, and Silhouette Coefficient [26].

The fuzzy method has been implemented for numerous techniques used for image segmentation. The reason behind the popularity of fuzzy image segmentation is its widespread applications in numerous areas i.e., fuzzy set theory, genetic algorithms, neural networks, computer vision, pattern recognition, and image processing [27]. EDAS is a mechanism of fuzzy logic introduced by Ghorabaee et al. [28], called Evaluation Based on Distance from Average Solution (EDAS). It is a novel scheme of the Multiple Criteria Decision-making Method (MCDM) that is used for the classification of inventory and one of the techniques for Multiple Criteria Decision-making [29]. One of the most popular techniques of FL is the EDAS method used for the ranking of algorithms to identify the best possible technique based on execution time, speed, and accuracy. One of the contributions of this paper is to explore the use of EDAS in the context of image segmentation.

The segmentation of color images is an extremely challenging task due to complexities associated with finding the number of clusters and the cluster centroids. Therefore, automatically finding the number of clusters and the centroids using an adaptive technique of unsupervised color image segmentation by applying clustering could prove beneficial, as we will demonstrate empirically in this paper. To fulfill the clustering requirements, recent efforts have carried out research using clustering methods such as K-means [30], modified K-means [31], and Ant Colony Fuzzy C-means Hybrid Algorithm (AFHA) [32].

The primary information about the number of clusters is unknown in real applications of color images [33]. The subjective information provided by human intervention in the previous methods highly degrades the clustering results of the color images. One of the important criteria for adopting clustering methods to the images is the provision of initialization parameters, i.e., number of clusters and the cluster centroids. The quality of the segmented images highly depends upon the parameters of initialization. The process of determining the initialization parameters is a challenging task, especially if image features are to be preserved.

In this study, we present a novel adaptive scheme that comprises a region splitting and merging technique and a K-means clustering method. A color image with RGB pixels is the combination of several homogeneous regions that have various intensity ranges of each RGB color channel. The region splitting and merging technique determines the peaks along with the consistent intensity level of each color channel. In the next step, the adjacent peaks of RGB pairs are combined to prevent the increasing number of clusters, which will lead to over-segmentation and to a loss in the classification accuracy. The combination of the RGB pairs referred to as the parameters for initialization, whereas the number of RGB pairs is considered as the clustering numbers. In the subsequent step, the K-means method performs the clustering of pixels in images by adopting the aforementioned parameters for initialization. For evaluating the advantages of our proposed method, we have made several comparisons with other works using the Berkeley Segmentation Dataset and Benchmark (BSDS500).

The rest of the article is organized as follows: First, a comprehensive related work about the technique proposed in this article is explained. Next, details about the architecture of our method are described. The subsequent sections focus on detail analysis and discussion of the experimental results, as well as the details of the used dataset; a qualitative and quantitative comparison of the results with respect to other existing methods is also presented. The final section discusses the conclusion along with the future scope of the presented technique.

## Related work

Image segmentation is an active area of research in image analysis. It encompasses various image processing techniques that seek to partition the image into multiple objects to improve task such as image analysis and feature extraction [34]. Image segmentation requires expert knowledge and guidance to some extent [35]. It divides neighboring pixels into smaller regions to analyze the Object of Interest (OOI) [36]. For a detailed account of region segmentation, the interested reader is redirected to other excellent surveys [20]. The segmented region is created by a combination of pixels that are connected with some type of distance metric using color and texture features of the image. Image segmentation can be explained more formally as given in [37] as follows: suppose *F* denotes the combination of pixels and *P*() is uniformity (homogeneity) predicate of connected groups of pixels that are already well defined, then then the segmentation task implies the partition of the set *F* into a cluster of regions and subsets (*S*_1_, *S*_2_, ⋯ *S*_*n*_) such that,

$$\begin{array}{c} \overset{n}{\underset{i=1}{⋃}}S_{i}=F\;with\;S_{i}\cap S_{j}=\Phi ,i\neq j \end{array}$$ (1)

The predicate of uniformity *P*(*S*_*i*_) = *true*∀ regions, (*S*_*i*_) and *P*(*S*_*i*_ ∩ *S*_*j*_) = *false* if *S*_*i*_ is adjacent to *S*_*j*_ as mentioned in Eq (1). According to the definition, an image that is to be segmented can be analyzed by an inter-region discrepancy between segments and intra-region homogeneity within a segment.

A comprehensive definition of image segmentation is presented in [38, 39]. According to [39], regions should be uniform, the boundaries of the regions must be simple, not ragged and adjacent regions must have a significant difference according to the considered uniformity criteria. In classical clustering algorithms such as K-means and Fuzzy C-means (FCM), objects are categorized into different classes based on similar attributes of the data objects [40–43]. The K-means technique is considered one of the simplest methods with a fast convergence [43]. Conversely, the FCM method does not consider the image contents and thus, it has high susceptibility to additive noise and it is not capable of handling the noisy images [44]. This technique also involves complex calculations and mostly leads to over-segmentation. Likewise, the conventional approach of K-means requires prior information about the images, such as the number of clusters and the initial centroid information of clusters in advance. Predefined parameters provided by the users highly influence the clustering results as the user has no prior knowledge about the number of clusters. Therefore, many studies have introduced adaptive techniques for cluster initialization to cope with these issues. For instance, Fukunaga and Hostetler [45] developed the Mean-shift (MS) algorithm which does not require predefined knowledge about the number of clusters nor any other kind of parameterization.

Most recently, the AFHA presented in [32] is an adaptive unsupervised clustering algorithm. AFHA is the combination of two techniques: Ant System and Fuzzy C-means algorithms. Ant System [46] identifies the compact and distinct clusters. Yu et al. mentioned in [32] that AFHA is a good approach in comparison to X-means [47], mean-shift (MS) [45] and Normalized cut [48]. Another unsupervised adaptive scheme for image segmentation is the modified K-means (MKM) algorithm proposed in [31]. This method is the modified version of the standard K-means clustering technique known as Bisecting K-means [49]. The MKM scheme repeatedly bisects clusters into subcategories until the desired number of K clusters is produced and the inter-cluster similarity is lower than the predefined threshold. The overall output of MKM highly suffers from a similar thresholding index. On the other hand, another hybrid based adaptive clustering algorithm for image segmentation was also introduced in [34], but its applicability is limited to gray-scale images.

An improved version of the AFHA algorithm was developed by Yu et al. [32] called Improved AFHA (IAFHA). IAFHA makes use of the Ant system algorithm to create the number of clusters and central points of the clusters. It takes a small amount (roughly 35%) of the total number of pixels. This enhancement in IAFHA overcomes the computational complexity of the conventional AFHA, but it is highly suffers from a low performance ratio.

The Evaluation Based on Distance from the Average Solution (EDAS)-based schemes are applied for the ranking of referenced techniques in this study. Some recent studies about numerous contributions of fuzzy EDAS scheme in various areas are summarized in Table 1.

**Table 1:** Fuzzy EDAS’s contributions in related research work.

Author	Title of study	Methodology-description
Peng et al. [50]	Algorithms for neutrosophic soft decision making based on EDAS, new similarity measure and level soft set	Developed three-level algorithms for solving the problems of a single-valued neutrosophic soft set by adopting the EDAS scheme.
Ilieva et al. [51]	Decision analysis with classic and fuzzy EDAS Modifications	Presented the L1 metric in EDAS approach for fixing some issues in MCDM problems to decrease time complexity.
Liang et al. [52]	An Integrated EDAS-ELECTRE Method with Picture Fuzzy Information for Cleaner Production Evaluation in Gold Mines	Suggested the method about four-level degrees of membership with PFNs (picture fuzzy numbers) to assess the production of cleaner for gold-mines.
Li et al. [53]	Linguistic Neutrosophic Multi-criteria Group Decision-Making Approach with EDAS Method	The proposed method developed the MCGDM (Multi-criteria Group Decision-Making) technique that is based on the EDAS for resolving the collective management of neutrosophic problems.
Stevic’ et al. [54]	Evaluation of Suppliers Under Uncertainty: A MultiphaseApproach Based on Fuzzy AHP and Fuzzy EDAS	The FuzzyAnalytic Hierarchy Process is proposed to select and evaluate the suppliers and also for the analysis of the Fuzzy EDAS method.”
Mehmood et al. [55]	A Trust-Based Energy-Efficient and Reliable Communication Scheme (Trust-Based ERCS) for Remote Patient Monitoring in Wireless Body Area Networks	The presented scheme is for a reliable communication method in order to maintain the privacy of WBAN (Wireless Body Area Network). The scheme is evaluated by EDAS ranking technique and declared on top rank.

Clusters in K-means are obtained by minimizing the sum of squared distances between objects and their resultant cluster centroids [56–60]. The most important concern in the K-means algorithm is to assign every cluster to *K* centroids and place the *K* central points as far as possible from other centroids. The data point is assigned from the dataset to the adjacent centroid. If no data points are left over, the first step is completed [60, 61]. New *K* central points are recalculated in the second iteration by following the procedures of the first step. The data points are allocated to its proximate new centroid [60, 62]. The locations of *K* centroids are changed with the assignment of new data points. The squared objective function is minimized by using K-means and is computed using the following expression [56, 63].

$$F=\sum_{j=1}^{y}\sum_{i=1}^{k}\cdot {\left\|y_{j}^{\left(d\right)}-\mu_{k}\right\|}^{2}$$ (2)

where $y_{j}^{\left(d\right)}$ denotes the *jth* data point of the cluster, *μ*_*k*_ identifies the *kth* centroid. Thus, Eq (2) computes the distance of similar objects to their respective cluster groups.

The classical K-means outperforms existing fuzzy methods on M-Fish segmentation, but the major issue in standard K-means is a lack of quality in segmentation of color images. The K-means approach suffers from local minima in the iterative procedure of optimization. It requires good initialization conditions, i.e., the number of pixel clusters required for image segmentation and the initial values of cluster centroids.

In this paper, we propose a novel cluster-based technique for color image segmentation, which can automatically recognize the central points of clusters by searching the RGB pairs accurately without requiring prior knowledge. More precisely, the RGB pairs improve the accuracy of the existing aforementioned algorithms by determining the automatic initialization parameters for standard K-means and the amount of RGB pairs acts as the number of clusters. Additionally, an efficient EDAS rank-based approach is presented for the ranking of proposed as well as reference algorithms and returned to the list of ranks by declaring the proposed technique on top rank.

## Proposed technique

The traditional K-means method is highly affected by the parameters of initialization for the central points of clusters and the number of clusters. Hence, the segmentation of color images by using K-means highly depends upon the parameters of initialization. Generally, selecting the suitable initialization method involves a laborious job by performing an extensive range of experiments. The repetitive process is adopted to perform a certain number of experiments to obtain robust initialization conditions. Therefore, a lengthy process is required to achieve the initialization approach for the K-means algorithm.

**Fig 1:**
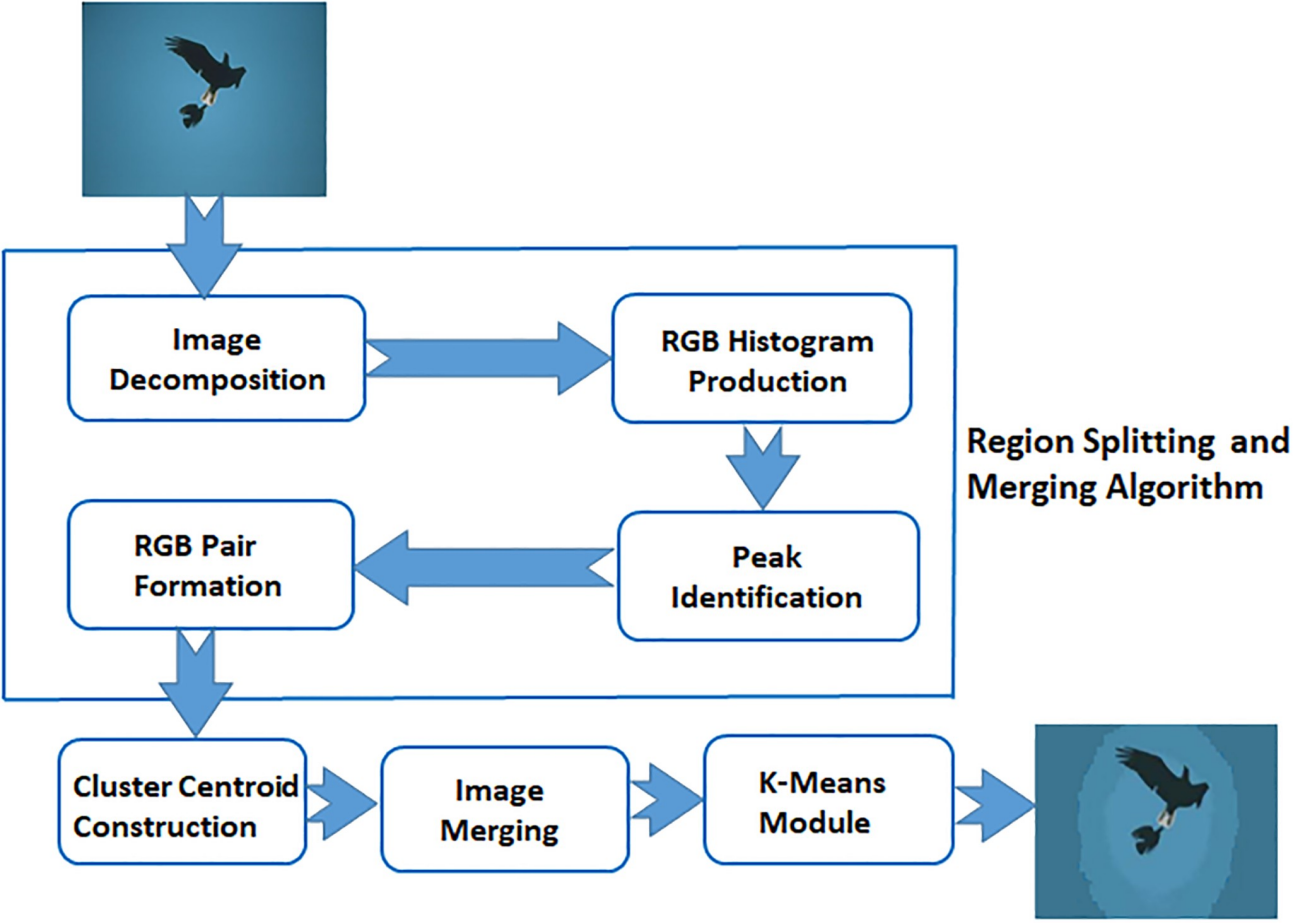
Block diagram of the proposed technique.

In this work, we propose to use a region splitting and merging technique as an optimized initialization approach that determines the number of clusters and centroids of clusters in a non-parametric and adaptive manner. In comparison with the extensively utilized random initialization process, the initialization process based on the region splitting and merging technique yields adaptive initialization parameters. It identifies the cluster numbers and clusters central points based on both the global and local information produced by the histogram of the input sample image. More specifically, every single pixel in a color image with red, green, and blue representation contains a combination of the RGB color-channel intensity values. The basic aim of the proposed technique is to improve a few limitations of the classical K-means clustering algorithm. The block diagram of the proposed technique is illustrated in Fig 1 that summarizes the rationale of the proposed technique in more detail. In this study, the region splitting and merging technique is elaborated in detail in the next section.

**Algorithm 1** New Centroid Calculation Algorithm

1. Calculate the new distance between the number of clusters

$$\begin{array}{c} d:=\sum_{\forall sj\in c}^{n}(\overset{m}{\underset{y_{i}}{⋃}}\frac{R_{n}G_{n}B_{n}}{m})\in \omega^{\prime } \end{array}$$ (3)

where *m* identifies the number of cluster, *ω*′ and *c* denotes the centroid intensity and centroid of cluster respectively. *R*_*n*_ *G*_*n*_ *B*_*n*_ are the pixel set assigned to the *ith*, *jth* and *kth* cluster sets, *yi* is the number of pixels assigned to *ith* cluster, and *sj* is the *ith* pixel in that cluster as illustrated in Eq (3). Calculate the new central points for each group of clustering intensities.

2. Calculate new data points of centroids for each group of clustering intensities in Eqs (4) and (5):

$$\begin{array}{c} \begin{array}{cc} \omega_{i}^{{}^{\prime }}\omega_{j}^{{}^{\prime }}\omega_{k}^{{}^{\prime }}:= & [\frac{1}{m_{i}}\sum_{s_{i}\in c_{i}}^{}R_{i}G_{i}B_{i}]\\ & \left[\frac{1}{m_{j}}\sum_{s_{j}\in c_{j}}^{}R_{j}G_{j}B_{j}\right]\left[\frac{1}{m_{k}}\sum_{s_{k}\in c_{k}}^{}R_{k}G_{k}B_{k}\right] \end{array} \end{array}$$ (4)

$$\begin{array}{c} \begin{array}{cc} = & [\frac{1}{m_{i}m_{j}m_{k}}\sum_{s_{i}\in c_{i}}\sum_{s_{j}\in c_{j}}\sum_{s_{k}\in c_{k}}\left(R_{i}G_{i}B_{i}\right)\left(R_{j}G_{j}B_{j}\right)\\ & \left(R_{k}G_{k}B_{k}\right)]\forall c_{i}\neq c_{j}\neq c_{k} \end{array} \end{array}$$ (5)

### Region splitting and merging technique

**Fig 2:**
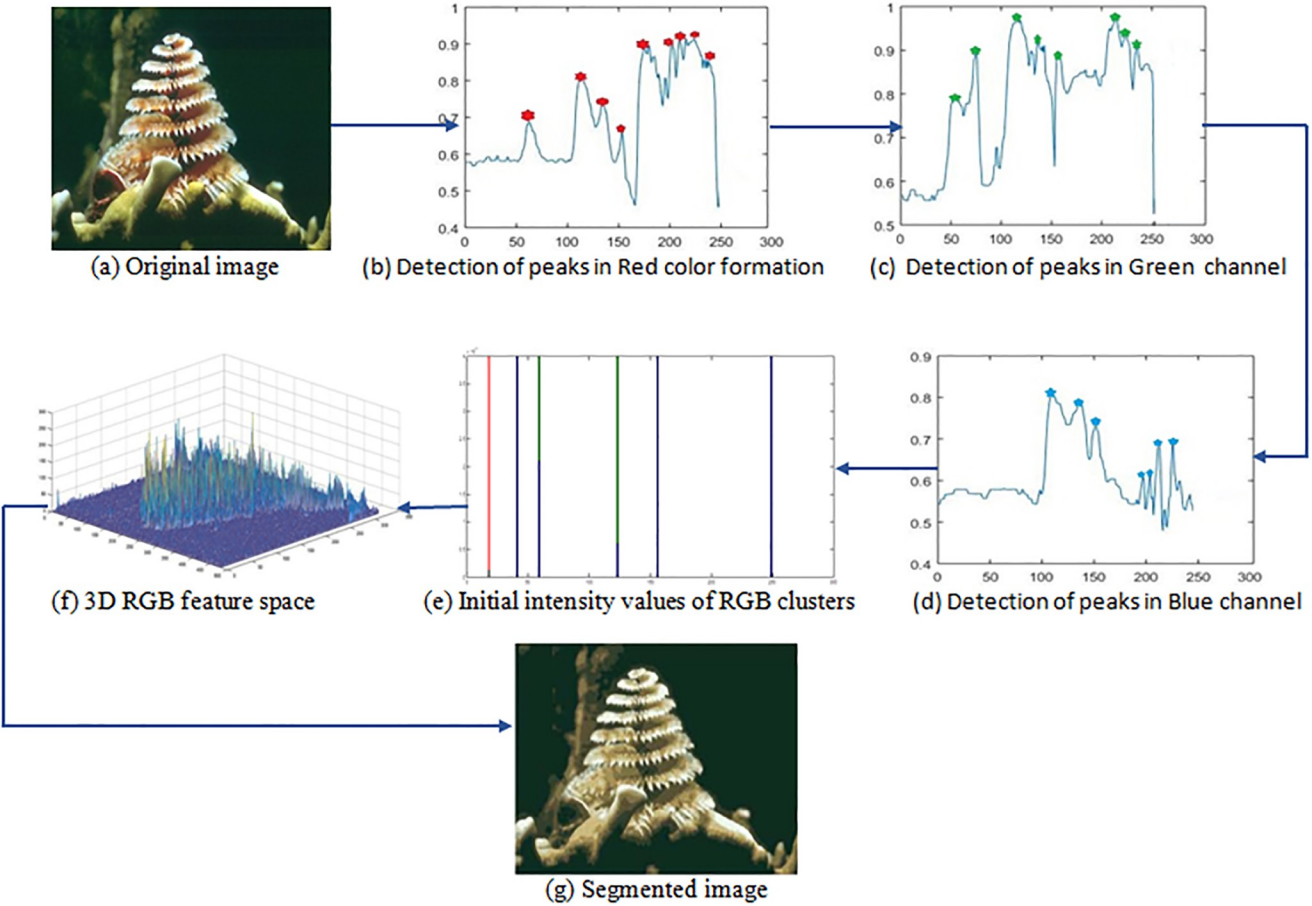
Phases of the presented technique.

In the proposed technique, the clustering integrity of the conventional K-means algorithm has been significantly improved for color-based image segmentation. The required number of clusters and central points of clusters perform the initialization scheme for the K-means cluster method in a more robust and accurate way compared to other random techniques for initialization. The region splitting and merging technique requires less laborious work and determines an accurate initialization condition and improving the overall accuracy over the baseline K-means method. The region splitting and merging technique is implemented as follows:

1. The method first analyzes the complete image and then produces the salient peaks for RGB color-channel histograms by identifying the intensities of channels concerning the maximum occurrences of points among the levels of the neighboring intensities. The peaks identified by the RGB color-channel histograms are represented using asterisks while the pits are unmarked as shown in Fig 2(b)–2(d), respectively.
2. Then, it classifies RGB pairs by detecting the missing color intensity values to identify the peaks of each homogeneous region lies in the already identified color-channel intensity ranges of each homogeneous region. The peaks identified by the red channel are denoted by asterisks in Fig 2(b).
3. Afterwards, a distance measure is calculated by using the Manhattan distance among all RGB pairs by applying the following equation. The GB(green and blue) color-channel peaks detection about a particular R(red) color-channel peak in Fig 2(b) is illustrated in respectively Fig 2(c) and 2(d):
  $$\begin{array}{c} D\left(c_{k},c_{l}\right)=|R_{k}-R_{l}|+|G_{k}-G_{l}|+|B_{k}-B_{l}|,\;\;\;\forall \;\;k\neq l \end{array}$$ (6)
  where 1 ≤ *k* ≤ *M*, 1 ≤ *l* ≤ *M*, *M* and *N* represent the number of RGB pairs, *R*_*k*_, *G*_*k*_, *B*_*k*_ are the intensities of the red, green, and blue color channels of the *k*th RGB pairs and *R*_*l*_, *G*_*l*_, *B*_*l*_ are the intensities of the red, green, and blue color channels of the *l*th RGB pairs respectively.
  (**Note**: The Manhattan distance measures better distance similarity compared to Euclidean distance because Manhattan has the stability characteristic of visual color similarity, whilst the latter produces a broader variation of the same color).
4. Calculate the minimum distance between the two adjacent cluster centroids.
5. Then, it calculates the new distance between the number of clusters and the new central points for every clustering group by using Eqs (5) and (6) respectively.
6. The RGB histogram is analyzed and the change in distance is calculated along the average change between peaks. The peak points that are above the average were allocated as the initial values of clusters, which are used in the clustering method, and also the number of peaks were assigned to the standard K-means clustering technique. The initial points of clusters are illustrated in Fig 2(e) and the number of clusters determined after completing the Algorithm 2, which provides the initialization condition for the standard K-means clustering algorithm.
7. Finally, it segments the pixel of clusters into proper regions by preserving the original RGB color features of the image is in Fig 2(f).

The proposed adaptive algorithm calculates the new cluster centroid in Algorithm 1 and also finds the initialization condition by using the region splitting and merging technique for standard K-means is given in Algorithm 2. The final segmented image is observed as the original foreground features of the coral image obtained by an adaptive approach is shown in Fig 2(g).

**Algorithm 2** Proposed Segmentation Algorithm

**Input**: Color image *CI*

**Output**: Optimal segmented color image *CI_seg_*

1. Cluster dimension ← *k*, cluster initialization = *ϕ*

2. **for** each cluster **do**

3. Find *d* = ∀ pixels & *ω*′*by* calling new centroid calculation Algorithm (1).

4. **end for**

5. **for** *j* = 1:255 //Record peak(*pk*) and pit(*pt*) values in the intensity distribution

6. **If** the values are decrease from increase, **then**

7. record *pk* values otherwise record *pt* values

8. **end If**

9. **end for**

10. Exclude the values which are less than the mean by vertical scanning of histogram

11. **for** *i* = 1 : *l*(*pks* and *pts*) *j* = 1:*d*(pks and pts) // Calculate the mean values, where *d* denotes dimension.

12. **If** the difference among the each and every values is greater than average number **then**

13. calculate only *pks* at great values of peak

14. **end If**

15. **end for**

16. Exclude the values which are less than the mean by horizontal scanning of histogram

17. Positions = find out the positions of great peaks at the histogram

18. **for** *j* = 1:*d* (Positions) **do** // *d* identifies dimension

19. Total = absolute distance of each high peak value with the mean greater than each pit value

20. **end for**

21. $Meanvalue=\frac{\text{total}\;\text{measure}}{\text{length}\;\text{of}\;\text{clustering}\;\text{positions}}$ // Calculate mean value of cluster

22. **for** i = 1:length (Positions)

23. *j = 1:d* (Points)

24. **If** the absolute measures of each elements of high peak values with the mean greater than pit values, **then**

25. identify the high clustering points

26. **end If**

27. **end for**

28. *k* = dimension (cluster result), initialization *(k)* = cluster result

29. **return** *CI_seg_*

## Data, experimental results and evaluation

In this study, the latest version of the Berkeley Segmentation Dataset and Benchmark (BSDS500) [64, 65] has been used. The images in the entire dataset are based on numerous classes, i.e., animals, airplanes, humans, natural scenes, trees, ships, and beaches, etc. which are considered as some of the most challenging samples for segmentation tasks. The dataset also includes ground-truth images annotated by 30 different individuals. Each image in the dataset has an average of 5 to 6 referenced images and is considered the best dataset for carrying out segmentation comparisons. The BSDS500 dataset and benchmark are used for evaluating the presented scheme due to the fact that comprises various categories, with the presence of many human-generated images. In our work, the database has been categorized into 10 various sub categories according to some specific image content for further evaluation of segmentation in preserving the original details of the input sample images.

The proposed technique is compared with the state-of-the-art methods for validating the results of the proposed segmentation technique and those in the state of the art methods. Next, the proposed scheme is evaluated by applying qualitative and quantitative measures of the proposed technique with referenced schemes. The results of qualitative and quantitative evaluation and EDAS based ranking verified that the proposed approach effectively improves the cluster integrity and shows a promising reduction in classification error of the segmented images.

### Qualitative based evaluation of the clustering results

**Fig 3:**
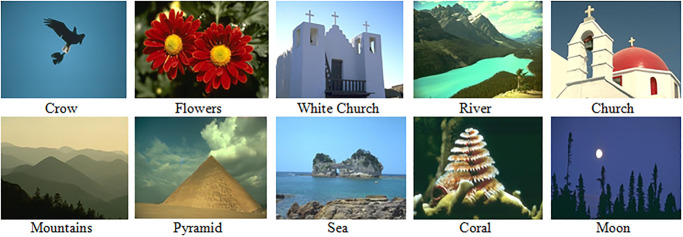
Original test images from BSDS500 dataset [64, 65].

**Fig 4:**
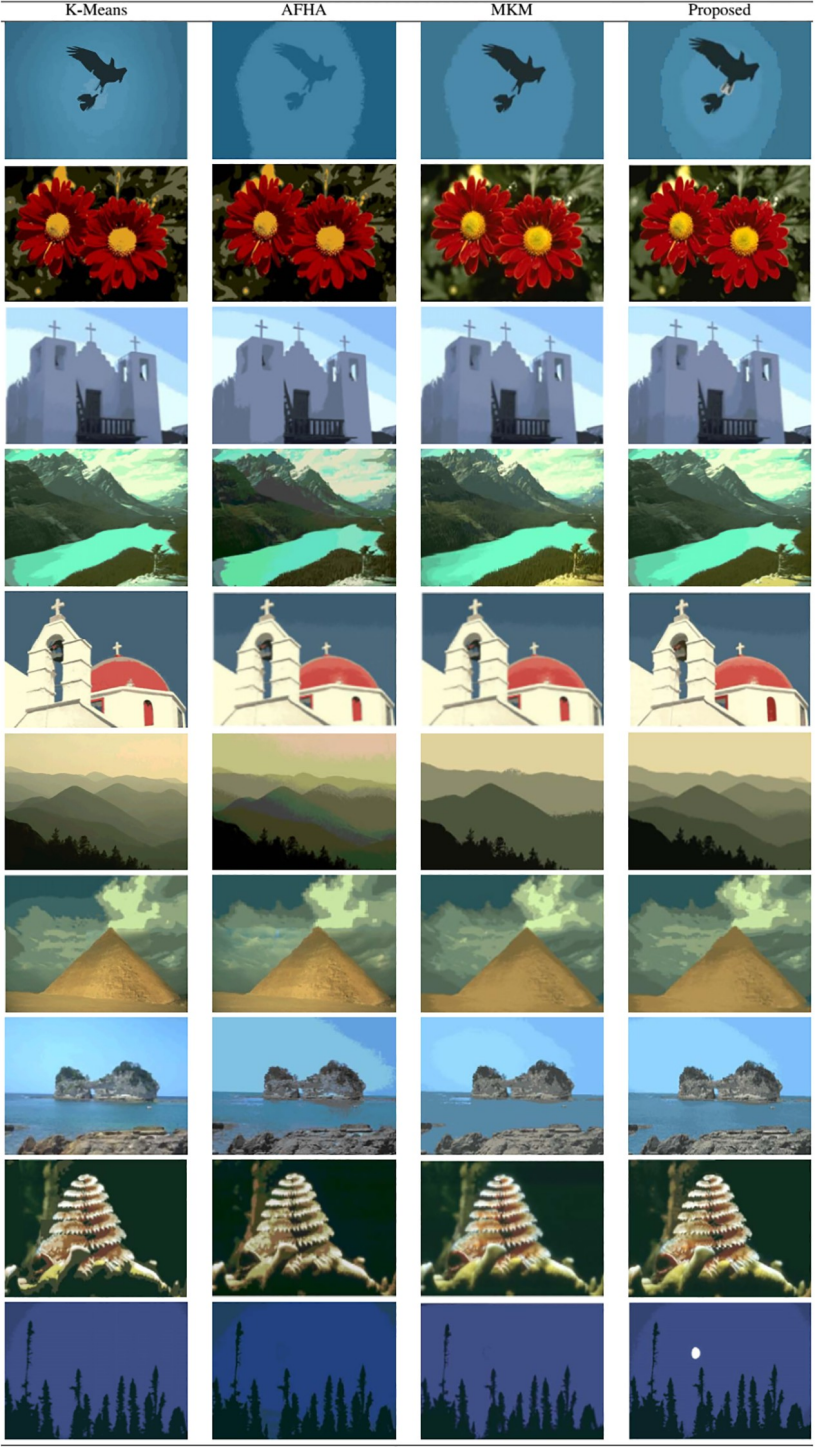
Results comparison of test images from BSDS500 dataset.

The comparative assessment of the results of each algorithm for some test images from the BSDS500 dataset in Fig 3 is illustrated in Fig 4. By discerning the visual detail of the resultant images of reference schemes, the proposed method produced optimal segmented images compared to other approaches and reflected the original detail of the images. In the example of the Bird image in Fig 4, K-means and AFHA produced a large number of clusters in the sky area and MKM has the result of the under-segmented background and also generated misclassification error by mistakenly assigned the white feathers of the Bird into the blue pixels of the background. On the other hand, the proposed scheme produced optimal segmentation, which can be observed in the foreground features in the sky region.

By noticing the details of the images of Flowers and White Church illustrated in Fig 4, the K-means and MKM generated almost the same results except for the fact that the K-means result had a noisy background with tiny clusters in the White church image. The results of both algorithms highly suffered from classification errors in the Flowers image such that the yellow pixels are mistakenly assigned to white pixels. The AFHA and the proposed scheme have a similar segmented background (sky) of the White Church image, while MKM and presented approach have similar results in the Flowers image. MKM produced over-segmentation of the foreground region in the Flowers image while under-segmented the White Church image. In the example of River image, K-means and AFHA produced homogeneous results with an obvious classification error by assigning the cyan color of a river to the ground region of the tree line in the lower right-hand location along with the higher number of clusters, resulting in over-segmentation. Some pixels of the ground are mistakenly assigned as the river pixels. The MKM generated an over-segmented result as many tiny clusters are created of the river water. Therefore, the presented method addressed the over-segmentation and false classification problems by following a better clustering process and retained the image details in the sample River images.

For the images of Church, Mountains, and Pyramid, K-means had yielded misclassification results in the foreground area, while AFHA and MKM had under-segmentation results. The proposed technique retains the trade-off concerning the preservation of image features and to produce uniform regions for all three images. K-means and MKM produced many tiny clusters in the background region of the Sea image, while the segmented images performed by AFHA created misclassified and over-segmentation regions.

In the example of the Coral image, K-means, AFHA, and MKM generated false classification results by insufficient assignment of object pixels and numerous tiny pixels appeared in the background. The visibility of the Moon image in Fig 4 evidence that K-means, AFHA, and MKM produced false classification error by assigning background pixels to the moon region and missing important information, leading to an incorrect segmentation of the object moon. AFHA results in the production of less homogeneous background by inaccurately assigning object pixels in the sky region and also over-segmented region, whilst tiny clusters were produced by K-means. MKM created a more homogeneous background, but it missed the image detail information in image Moon, while the proposed approach preserved the image detail and successfully recovered false classification error by producing accurate clusters that lead to optimal segmentation.

It can be concluded from the comparison of results in Fig 4 that the proposed technique generates an optimal number of segments in the background in all the sample images. In all results, AFHA has resulted in under-segmentation and false classification in almost all sample images. Conversely, K-means and MKM yielded over-segmented images; K-means also produced a considerable number of tiny clusters in the background.

### Quantitative based evaluation of the clustering results

There are numerous benchmarks mentioned in the literature for the evaluation of image segmentation methods. The two major categories of evaluation results for the segmentation of images are supervised evaluation methods and unsupervised evaluation methods [66]. The supervised methods evaluate the segmented techniques by comparing the segmented results of images with the ground truth images and unsupervised methods analyze the characteristics of the segmented results with the human-generated images. In the supervised method, subjects are involved which affects the results and makes it time-consuming, whereas no subjects are involved in unsupervised methods that provide objective and quantitative results. For this reason, the unsupervised evaluation is adopted in this study.

#### Evaluation of image cluster number

The section examines the effectiveness of segmentation quality depending upon the result of the cluster number. There is a trade-off between the generated number of cluster and the segmentation quality of homogeneous regions, whereas inadequate clustering numbers produced in the process of segmentation could result in misclassification errors, as displayed in the Bird, Mountain, Coral, and Moon images as depicted in Fig 4 above. A more reliable segmentation can be achieved by obtaining further homogeneous pixels while observing a sound clustering number. As it can be observed in Table 4, both AFHA and the proposed scheme yield a fewer number of clusters compared to K-means and MKM methods for the three images of Flowers, River, and Sea. Hence, the AFHA and proposed technique both lead to better segmentation results; for instance, we can observe that there are less clusters in the images of Flowers, River, and Sea. For the Church image, the proposed and classical K-means methods produce fewer clusters by optimized segmented images with greater homogeneous regions as compared to MKM and AFHA techniques. The AFHA method mistakenly assigned considerable pixels to the sky (i.e. background) while producing the segmented regions in the Church image. Moreover, Table 2 clearly shows that the segmentation performed by AFHA and MKM techniques on the Bird, Mountains, and Moon images produced segmented regions with better homogeneity while finding a fewer number of clusters compared to K-means and the proposed method. Conversely, a significant number of pixels are falsely assigned to the sky regions in the images segmented by K-means, MKM, and AFHA. K-means produced over-segmented regions in the images of Bird, Mountain, and Moon (Fig 4). The proposed technique successfully avoided these false classification errors and returning the accurate number of cluster. For the images of Pyramid and Coral, AFHA and proposed method produce a fewer number of clusters with greater homogeneity in the segmented image compared to K-means and MKM, which yield higher clusters. Similarly, for the White Church image, the proposed scheme produces a compact number of clusters while preserving the segmentation quality in the segmented image than the K-means, AFHA, and MKM methods.

**Table 2:** Number of clusters generated for various images using different segmentation’s techniques.

Methods	Images									
	Bird	Flowers	White Church	River	Church	Mountains	Pyramid	Sea	Coral	Moon
K-means	10	255	100	70	10	15	80	17	220	9
AFHA	4	**6**	50	17	25	11	**60**	**7**	**10**	5
MKM	**3**	248	90	55	25	**4**	66	12	191	**4**
Proposed	6	**6**	**47**	**15**	**7**	8	62	**7**	15	6

**Note**: The bold entries indicate the best result achieved among the different methods for a given sample image.

#### MSE based evaluation

The MSE (Mean Squared Error) is a benchmark that measures the quality of clusters. The MSE metric was used for evaluating the segmentation results of various existing methods and the proposed technique, using the sample images from the BSDS500 dataset. The Mean Square Error is defined as follows in Eq (7):

$$\begin{array}{c} MSE=\frac{1}{N}\sum_{j=1}^{M}\sum_{i\in S_{i}}^{}{∥x_{i}-c_{j}∥}^{2} \end{array}$$ (7)

where *N* denotes the total number of pixels in the image, *M* identifies the clustering numbers produced during clustering procedure, *S*_*j*_ indicates the set of pixels belonging to *j*th cluster, *c*_*j*_ specifies the feature vectors of the *j*th central points of clusters and *x*_*i*_ states the feature vectors of the *ith* pixel belonging to *jth* data points. Consequently, MSE measures the average deviation between the number of clusters and cluster centroids.

The results of the MSE analysis using K-means, AFHA, MKM, and the proposed technique are summarized in Table 3. As it can be observed from this comparison, the proposed technique yielded the lowest MSE values for most of the images except the Pyramid sample as compared to other approaches. The results produced by proposed technique for sample images of Birds, Moon, White Church, River, Flower, Church, Mountains, Coral, and Sea have the lowest MSE while K-means, AFHA, and MKM result in the highest MSE for all these images. AFHA results in the lowest MSE for the image Pyramid. Our findings indicate that the proposed technique results in the lowest values of MSE i.e. 93% of all the sample images. Overall, the experiment applied on 200 images, K-means, AFHA, and MKM results of 4%, 3.5%, and 5% respectively whereas the proposed approach results in 82.5% along with the lowest value of MSE. Thus, the lowest MSE values produced by the proposed approach have better cluster quality compared to other techniques and also visually investigated in Section 4. The ability of the proposed technique to yield consistently lower MSE values verifies that it produces clusters with minimum distortion than other methods.

**Table 3:** MSE based comparison of clustering quality for various initialization techniques.

Methods	*MSE*(*1.0_*e*_ + 2)									
	Bird	Flowers	White Church	River	Church	Mountains	Pyramid	Sea	Coral	Moon
K-means	4.2532	4.0142	3.3546	4.2511	3.0342	4.2135	3.5121	3.4360	2.9812	5.1643
AFHA	1.8605	3.2542	2.2021	4.9352	1.3334	4.1611	**3.4452**	2.5611	2.1571	1.8203
MKM	2.6511	2.2541	2.3214	3.8245	1.7129	3.2431	4.9454	1.5732	3.0152	3.0122
Proposed	**1.0324**	**1.7621**	**2.1011**	**3.7542**	**1.3224**	**1.7934**	4.0112	**1.2342**	**2.0761**	**0.1723**

**Note**: The bold entries indicate the best result achieved among the different methods for a given sample image.

### Ranking based evaluation of the clustering results

In this research, the fuzzy logic-based EDAS method is used for evaluating the ranking of the proposed scheme compared to the reference algorithms, in terms of segmentation integrity. At present, the Evaluation Based on Distance from Average Solution is adopted for MCDM. In this study, the authors presented the EDAS scheme to accumulate cross-efficient values. The aggregate of Appraisal Scores (AS) can be measured for ranking of reference schemes to calculate the positive distance from average solution (PDA) and negative distance from average solution (NDA).

In the below Table 4, the images are considered as the criteria of MCDM.

**Step 1**: Calculate the solution of the average value of all images in Eq (8);
  $$AV={\left[AV_{n}\right]}_{1\times q}$$ (8)
  where
  $$\begin{array}{c} AV_{n}=\frac{\sum_{i=1}^{k}X_{mn}}{k} \end{array}$$ (9)
  The above step determines the image segmentation as per the MCDM approach. The aggregate about the calculation of Eqs (8) and (9) can be obtained as the average value for every image.
**Step 2**: This step of the EDAS calculates positive distances from average PDA in Eqs (10), (11) and (12) as given below:
  $$PDA={\left[PDA_{mn}\right]}_{q\times q}$$ (10)
  If the *n_th_* criterion is more beneficial then
  $$\begin{array}{c} PDA_{mn}=\frac{Maximum(0,\;\;(A_{V_{n}}-X_{mn}))}{A_{V_{n}}} \end{array}$$ (11)
  and if non-beneficial then the given equation will be changed as below:
  $$\begin{array}{c} PDA_{mn}=\frac{Maximum(0,\;\;(X_{mn}-A_{V_{n}}))}{A_{V_{n}}} \end{array}$$ (12)
  The calculated results are given in Table 5:
**Step 3**: This step of the EDAS calculates negative distances from average NDA using Eqs (13), (14) and (15) as follows:
  $$NDA={\left[NDA_{mn}\right]}_{q\times q}$$ (13)
  If the *n_th_* criterion is more beneficial then
  $$\begin{array}{c} NDA_{mn}=\frac{Maximum(0,\;\;(A_{V_{n}}-X_{mn}))}{A_{V_{n}}} \end{array}$$ (14)
  and if non-beneficial then the given equation will be changed as below:
  $$\begin{array}{c} NDA_{mn}=\frac{Maximum(0,\;\;(X_{mn}-A_{V_{n}}))}{A_{V_{n}}} \end{array}$$ (15)
  where *PDA*_*mn*_ and *NDA*_*mn*_ denotes the positive distance and negative distance of *n_th_* Rated Algorithms from the average value with respect to *m_th_* Rating Images, respectively.
  The calculated results are given in Table 6:
**Step 4**: Weighted sum of *PDA*_*mn*_ for the Rated Algorithms in Table 7 as below in Eq (16):
  $$\begin{array}{c} SP_{m}=\sum_{n=1}^{k}y_{n}PDA_{mn} \end{array}$$ (16)
**Step 5**: Weighted sum of ND *A*_*mn*_ for the Rated Algorithms in Table 8 as below in Eq (17):
  $$\begin{array}{c} SN_{m}=\sum_{n=1}^{k}y_{n}NDA_{mn} \end{array}$$ (17)
  The result reflected in the table below:
**Step 6**: This step normalizes and calculates the scores of *SP* and *SN* for the Rated Algorithms as follows in Eqs (18) and (19):
  $$\begin{array}{c} NSP_{m}=\frac{SP_{m}}{maximum_{m}(SP_{m})} \end{array}$$ (18)
  $$\begin{array}{c} NSN_{m}=1-\frac{SN_{m}}{maximum_{m}(SN_{m})} \end{array}$$ (19)
**Step 7**: This step calculates the scores of *NSP* and *NSN* in order to get appraisal score *(AS)* for the Rated Algorithms given as follows in Eq (20):
  $$\begin{array}{c} AS_{m}=\frac{1}{2}(NSP_{m}-NSN_{m}) \end{array}$$ (20)
  where 0 ≤ *AS*_*j*_ ≤ 1.
  The *AS* is determined by the aggregate score of *NSP*_*m*_ and *NSN*_*m*_.
**Step 8**: Measure the appraisal scores *(AS)* in terms of decreasing order and then determine the ranking of rated algorithms. The best ranking algorithms have higher *AS*. Therefore, in the below Table 9, the proposed algorithm has the highest *AS*.
  The final rank results are represented in the table below:
  The ranking shows the proposed algorithm is the best out of three existing algorithms.

**Table 4:** Cross-efficient values.

Algorithms	Images									
	Bird	Flowers	White Church	River	Church	Mountains	Pyramid	Sea	Coral	Moon
K-means	17.694	97.284	109.877	107.752	40.817	14.222	10.529	85.214	100.371	18.578
AFHA	33.113	46.482	80.987	70.216	37.709	37.583	**30.290**	48.665	40.227	26.788
MKM	**0.899**	36.302	90.874	40.353	59.059	**3.288**	32.828	23.784	**6.188**	8.289
Proposed	4.654	**25.277**	**67.988**	27.457	13.793	9.183	37.195	**22.885**	35.165	**0.897**
$A_{V_{n}}$	5.123	18.667	31.793	**22.343**	**13.761**	5.116	18.691	16.413	16.541	4.959

**Note**: The bold entries indicate the best result achieved among the different methods for a given sample image.

**Table 5:** Analysis results of average *PDA*.

Algorithms	Images									
	Bird	Flowers	White Church	River	Church	Mountains	Pyramid	Sea	Coral	Moon
K-means	0	0	0	0	0	0	0	0	5.067	2.746
AFHA	0	0	0	0	0	0	0	0	1.431	4.401
MKM	0.824	0	0	0	0	0.768	0	0	0	0
Proposed	0.091	0	0	0	0	0.357	0	0	1.125	0.671

**Table 6:** Analysis results of average *NDA*.

Algorithms	Images									
	Bird	Flowers	White Church	River	Church	Mountains	Pyramid	Sea	Coral	Moon
K-means	2.453	4.211	2.455	3.822	1.966	1.779	4.633	4.191	0	0
AFHA	5.462	1.489	1.547	2.142	1.740	6.346	0.620	1.964	0	0
MKM	0	0.944	1.858	0.806	3.291	0	0.756	0.449	0.625	0.818
Proposed	0	0.354	1.138	0.228	0.002	0	0.989	0.394	0	0

**Table 7:** Analysis results of the aggregate *PDA*.

Criteria(W)	0.235	0.181	0.122	0.118	0.073	0.042	0.042	0.085	0.049	0.047	*SP*_*m*_
Algorithms	Images										
	Bird	Flowers	White Church	River	Church	Mountains	Pyramid	Sea	Coral	Moon	
K-means	0	0	0	0	0	0	0	0	0.249	0.130	0.379
AFHA	0	0	0	0	0	0	0	0	0.070	0.209	0.280
MKM	0.194	0	0	0	0	0.015	0	0	0	0	0.209
Proposed	0.021	0	0	0	0	0.033	0	0	0.055	0.032	0.141

**Table 8:** Analysis results of the aggregate *NDA*.

Criteria (W)	0.235	0.181	0.122	0.118	0.073	0.042	0.042	0.085	0.049	0.047	*SN*_*m*_
Algorithms	Images										
	Bird	Flowers	White Church	River	Church	Mountains	Pyramid	Sea	Coral	Moon	
K-means	0.578	0.764	0.301	0.453	0.143	0.076	0.198	0.358	0	0	2.875
AFHA	0	0	0	0	0	0	0	0	0.070	0.209	0.280
MKM	0.194	0	0	0	0	0.015	0	0	0	0	0.209
Proposed	0.021	0	0	0	0	0.033	0	0	0.055	0.032	0.141

**Table 9:** Ranking based analysis for four segmentation algorithms.

Algorithms	*SP*_*m*_	*SN*_*m*_	*NSP*_*m*_	*NSN*_*m*_	*AS*_*m*_	Ranking
K-means	0.379	2.875	1	0	0.511	**3**
AFHA	0.280	2.597	0.737	0.096	0.417	**4**
MKM	0.209	0.876	0.551	0.695	0.622	**2**
Proposed	0.141	0.307	0.373	0.893	0.633	**1**

### Evaluation of the execution time

Some of the experiments were performed and the execution time of the proposed technique is compared with K-means, AFHA, and MKM. The algorithms were executed on Intel^®^ Core TM m3-7Y32 processor with the smart cache of 4 MB, 3.00 GHz frequency and 8 GB memory, 1 TB Hard drive with Microsoft Windows 10. The execution time of each algorithm is reported in Table 10.

**Table 10:** Execution time comparison of different schemes for sample images.

Execution Time (sec.)										
Image	Bird	Flowers	White Church	River	Church	Mountains	Pyramid	Sea	Coral	Moon
Image size	481×321	481×321	481×321	481×321	481×321	481×321	481×321	481×321	481×321	481×321
K-means	17.694	37.2846	95.8772	67.7522	**13.8177**	14.2223	40.2909	55.2143	50.3712	18.5784
AFHA	3.3113	46.4826	80.9876	70.2162	27.7095	27.583	**30.2909**	48.6654	40.2275	26.7884
MKM	3.8995	36.3027	90.8748	40.3534	30.1059	**3.2881**	32.8289	23.7849	**26.1888**	8.8977
Proposed	**2.6542**	**25.277**	**67.988**	**27.4573**	**13.7938**	4.1832	**30.1956**	**22.8854**	**26.1658**	**7.2894**

**Note**: The bold entries indicate the best result achieved among the different methods for a given sample image.

According to Table 11, it can be concluded that the proposed technique outperformed K-means, AFHA, and MKM by comparing time complexity per image. The rough estimate about the average time of the proposed technique per image is almost 19 seconds, which is the lowest time than K-means, AFHA, and MKM. The average complexity of time duration in seconds performed per image by K-means is round about 150 seconds, which is the highest time, and the average time of each image executed by AFHA and MKM is estimated at almost 63 seconds and 20 seconds respectively.

**Table 11:** Comparative analysis of different segmentation methods for 200 standard images.

Evaluating Parameter	K-means	AFHA	MKM	Proposed
Lowest MSE	4%	3.5%	5%	**82.5**%
Average time duration (sec.)	150s	63s	20s	**19s**
EDAS based ranking	3	4	2	**1**

**Note**: The bold entries indicate the best result achieved among the different methods for a given sample image.

From the results in Tables 10 and 11, we can assert that the proposed method yielded better results than K-means, AFHA, and MKM for execution time comparison and clustering quality. Henceforth, the proposed method is more applicable to segmentation applications in real-time scenarios. The summarized results reported in Table 11 indicate that the proposed technique consistently produces a better distribution of clustering with lower time execution in seconds. Thus, it is proved that the proposed technique is suitable to be adopted for unsupervised image segmentation.

## Conclusion

In this research, a novel adaptive approach for unsupervised color image segmentation was presented by applying region splitting and merging technique for optimizing the quality of segmented images. The proposed study optimizes the cluster centroids and determines the significant cluster numbers automatically for the initialization of the classical K-means algorithm. The proposed technique first determines the gradient change of distinct peaks of the RGB channel intensity. Next, the intensity values of RGB histograms are scanned and the highest peak intensity values are selected within the neighboring intensity ranges for each color channel. To observe the distinct peaks by further exploring each pixel histogram under the peak intensity values, which are to be selected to find RGB pairs. Those adjacent clustering points from the pairs are merged and the significant information of initialization parameters was assigned to the conventional K-means algorithm and in this way, finally, the image is segmented with a suitable number of uniform regions.

The proposed technique was compared with K-means, AFHA, and MKM by using image segmentation evaluation, and benchmarks. For further evaluation and clarification, the results of the EDAS ranking method showed that the proposed scheme ranked on top, MKM on 2nd, K-means, and AFHA on 3rd and 4th ranking respectively. Hence, the overall evaluation and experimental results clearly show that the proposed approach outperforms previous methods in terms of image segmentation quality and possible reduction of the classification error. However, the suggested technique has some challenges as it is not recommended for medical applications due to color degradation issues. Thus, in the future scope, we will work and enhance the colors of the resultant images to make it amenable to medical images.
